# Improved RA classification among early arthritis patients with the concordant presence of three RA autoantibodies: analysis in two early arthritis clinics

**DOI:** 10.1186/s13075-019-2079-4

**Published:** 2019-12-11

**Authors:** Cristina Regueiro, Lorena Rodríguez-Martínez, Laura Nuño, Ana M. Ortiz, Alejandro Villalba, Dora Pascual-Salcedo, Ana Martínez-Feito, Isidoro González-Alvaro, Alejandro Balsa, Antonio Gonzalez

**Affiliations:** 1 Experimental and Observational Rheumatology and Rheumatology Unit, Instituto de Investigacion Sanitaria-Hospital Clínico Universitario de Santiago (IDIS), 15706 Santiago de Compostela, Spain; 2 Rheumatology Department, Instituto de Investigación Hospital Universitario La Paz (IDIPAZ), 28046 Madrid, Spain; 3 Rheumatology Department, Hospital Universitario de la Princesa, Instituto de Investigación Sanitaria la Princesa (IIS-lP), 28006 Madrid, Spain; 4 Immuno-Rheumatology Department, Instituto de Investigación Hospital Universitario La Paz (IDIPAZ), 28046 Madrid, Spain

**Keywords:** Rheumatoid arthritis, Early arthritis, Disease classification, Autoantibodies, Rheumatoid factor, Anti-citrullinated protein antibodies, Anti-carbamylated protein antibodies

## Abstract

**Background:**

The patients with RA benefit from early identification soon after the first clinical symptoms appear. The 2010 ACR/EULAR classification criteria were developed to fulfill this need and their application has been demonstrated to be effective. However, there is still room for improvement. Therefore, we aimed to evaluate the potential of the concordant presence of RF, anti-CCP and anti-carbamylated protein antibodies to improve current RA classification among early arthritis (EA) patients.

**Methods:**

Data from the first visit of 1057 patients in two EA prospective cohorts were used. The serological scores from the 2010 ACR/EULAR criteria and the concordant presence of the three RA autoantibodies were assessed relative to a gold standard consisting of the RA classification with the 1987 ACR criteria at the 2 years of follow-up.

**Results:**

The concordant presence of three antibodies showed predictive characteristics allowing for direct classification as RA (positive predictive value = 96.1% and OR = 80.9). They were significantly better than the corresponding to the high antibody titers defined as in the 2010 classification criteria (PPV = 88.8%, OR = 26.1). In addition, the concordant presence of two antibodies was also very informative (PPV = 82.3%, OR = 15.1). These results allowed devising a scoring system based only on antibody concordance that displayed similar overall performance as the serological scoring system of the 2010 criteria. However, the best classification was obtained combining the concordance and 2010 serological systems, a combination with a significant contribution from each of the two systems.

**Discussion:**

The concordant presence of RA autoantibodies showed an independent contribution to the classification of EA patients that permitted increased discrimination and precision.

## Background

Advances in the management of RA have motivated an increasing interest in the early detection of the patients to ensure they obtain all the treatment benefits [1]. Accordingly, the classification criteria for RA were modified in 2010 by the ACR and the EULAR to include patients in the early phases of the disease [2]. This objective was partially attained as has been shown in multiple studies [3, 4]. Further improvements towards the early identification of RA patients have been sought in several areas including the presence of autoantibodies. Two of them, RF and anti-CCP antibodies, have already an important role in the 2010 ACR/EULAR RA classification criteria. The presence of any of these antibodies at titers over the upper limit of normal (ULN) is scored as 2 points or as 3 points if > 3 times the ULN. These scores represent a significant fraction of the 6 points required for RA classification. Other autoantibodies, including the anti-carbamylated protein antibodies (ACarPA), have been assessed, but none has significantly improved the classification obtained with RF and anti-CCP antibodies [5–7]. However, none of these evaluations has assessed the value of the concordant presence of the RA antibodies as a classifier. It is already known that the triple concordance is associated with extremely high specificity for RA, but analysis of its role in RA classification among EA patients has not been performed [8]. Therefore, the possibility that the concordant presence of RA autoantibodies could improve RA classification seemed worth to explore. Accordingly, we analyzed the data of the 1057 patients from the two Spanish EA cohorts included in Regueiro et al. [6] to assess the value of new criteria considering antibody concordance.

## Methods

The patient information and the antibody determinations have already been reported [6]. Briefly, EA patients from two prospective clinics in Madrid, PEARL [9] and IdiPAZ [10] recruited between July 2001 and December 2014 at PEARL and between January 1993 and December 2013 at IdiPAZ were studied. The entry criteria for the EA clinics were 2 or more swollen joints for less than a year and the absence of previous treatment with Disease-Modifying Anti-Rheumatic Drugs (DMARD). The patients were classified at the end of the 2-year follow-up according to the 1987 American College of Rheumatology (ACR) classification criteria [11]. This classification in RA and non-RA was used as the gold standard. The antibodies were determined in the sera from the first visit. The IgM-RF was assessed by nephelometry, whereas anti-CCP antibodies (ACPA) and anti-carbamylated protein antibodies (ACarPA) were determined by ELISA. The latter was a previously described home-made assay performed at Santiago de Compostela using in vitro carbamylated proteins from fetal calf serum as antigen [12, 13]. Briefly, we used FCS (F-7524, Sigma-Aldrich) at 4 mg/mL as a source of proteins for in-vitro carbamylation with 1 M KCNO, or with 1 M KCl as control, during 15 h at 37 °C. The efficiency and percentage of carbamylation were corroborated by HPLC in a Biochrom 30 amino-acid analyzer (Biochrom, UK). The ACarPA reactivity was assessed in duplicated diluted serum samples (50 μL at 1:50 dilution) incubated in separate Nunc MaxiSorp flat-bottom 96 well plates coated either with carbamylated or with native FCS (0.5 μg/well). IgG antibodies were detected using ALP-conjugated goat anti-human IgG (Jackson Immunoresearch Europe, UK). Reactivity to native FCS was subtracted from the reactivity to carbamylated FCS. A standard curve made with serial dilutions from a pool of positive sera was used to measure antibody titers in arbitrary units. The cut-off for positivity was set as the 98% specificity level obtained in 208 healthy controls.

The serological criteria according to the 2010 ACR/EULAR criteria were evaluated [2]. They comprised three levels: negative, low positive and high positive based in RF or anti-CCP titers. Negative titers were below the upper limit of normal (ULN), whereas low and high positive levels were defined in relation with 3 times the ULN [2]. In addition, the new criteria based on the concordance of the three autoantibodies were considered. All the levels were considered as categorical variables and only main effects were ascertained. The OR and their 95% confidence intervals were obtained from the logistic regression models. In addition, the model fit was assessed with the Nagelkerke *R*^2^, and the Akaike’s Information Criterion (AIC). The Nagelkerke *R*^2^ estimates the predictive power of the model as a proportional reduction in error variance. The AIC estimates the relative amount of information lost by any model. Therefore, the R^2^ measures increase with the predictive power of the model, whereas the AIC reaches lower values for the best models. Differences in AIC > 2 between two models are meaningful, whereas differences > 10 are interpreted as indicating essentially no support for the poorer model [14]. The impact of the different serological criteria on the overall classification (serological + non-serological criteria) was explored in the patients from PEARL, who featured all the required information. This exploration was done in two ways. The first consisted of changing the serological scores in the 2010 ACR/EULAR criteria. The second classified the patients with logistic regression that combined the non-serological and serological criteria applying cut-offs that were adjusted to obtain a constant sensitivity. The results of these classifications were expressed as specificity (true nonRA/observed nonRA patients), sensitivity (true RA/observed RA patients) and accuracy ((true nonRA + true RA)/all patients). The statistical tests were performed with R using the Jamovi application [15, 16].

## Results

The 1057 EA patients were stratified according to the serological component of the 2010 ACR/EULAR classification [2], or according to the presence of three, two, one, or none of the RA autoantibodies (Table 1). Therefore, the 2010 serological criteria classified the EA patients in three levels, whereas the concordance criteria produced four levels. There were clear discordances between the two stratifications. For example, the 54 patients presenting only ACarPA were scored 0 in the 2010 serological criteria and 1 in the concordance score, or the 46 patients at level 3 in the 2010 serological criteria that only presented 1 antibody (1Ab). However, the general distribution of frequencies was strongly correlated between the two systems (Gamma = 0.986, *p* < 10^−16^).

**Table 1:** Contingency table of the EA patients according to the 2010 serological criteria and the antibody concordance criteria

	Antibody concordance			
Scores	0Ab	1Ab	2Ab	3Ab
2010 criteria				
3	0 (0) ^a^	46 (11.7)	170 (43.1)	178 (45.2)
2	0 (0)	75 (67.6)	33 (29.7)	3 (2.7)
0	498 (90.2)	54 (9.8)	0 (0)	0 (0)

^a^Each cell of the table contains the number of EA patients and, between brackets, the percentage of the row total they represent

Analysis of the EA patient strata showed that the highest positive predictive values (PPV) were obtained with the concordance criteria (Table 2), both in the top and the medium levels corresponding to the concordance of 3 (3Ab) and 2 (2Ab) antibodies, respectively. The 96.1% PPV obtained with the concordance for the 3 antibodies would be sufficient to classify the patients as having RA. It was also noteworthy that the PPV obtained with the 1Ab level of the concordance criteria was remarkably similar to the PPV of the 2-points score of the 2010 serological criteria.

**Table 2:** Classification of the EA patients according to serological criteria

	2010 ACR/EULAR ^a^				Concordance		
Score	RA	Non-RA	PPV ^b^	Level	RA	Non-RA	PPV
3	350	44	88.8%	3Ab	174	7	96.1%
2	47	64	42.3%	2Ab	167	36	82.3%
–	–	–	–	1Ab	68	107	38.9%
0	129	423	23.4%	0Ab	117	381	23.5%

^a^The serological criteria defined in the 2010 ACR/EULAR RA classification criteria or by the concordance of autoantibodies

^b^*PPV* positive predictive value

The OR obtained separately with the 2010 ACR/EULAR and with the concordance criteria added a clear perspective of the high predictive power of the concordance of the 3 antibodies (OR = 80.9) relative to the observed with the high antibody titers in the 2010 criteria (OR = 26.1). Furthermore, the logistic regression model incorporating both criteria showed a significant contribution to the RA classification of the two (Table 3). The criterion with the largest weight was the concordance of the 3 antibodies. It was followed in decreasing order by the 3-points score, the concordance of 2 antibodies, the 2-points score and the presence of only 1 antibody. The two latter classifiers lacked a significant contribution. Therefore, we also tested the combined criteria after deleting the stratum corresponding to the presence of 1 antibody (Table 3).

**Table 3:** Analysis of the relative weights of the serological criteria and their combinations

	2010 ACR/EULAR^a^	Concordance	2010 + Ccd.	2010 + Ccd.’
Stratum	OR (95% CI) ^b^	OR (95% CI)	OR (95% CI)	OR (95% CI)
3	26.1 (18.0–37.8)	–	7.5 (3.3–17.0)	7.0 (4.0–12.2)
2	2.4 (1.6–3.7)	–	1.7 (0.8–3.7) ^c^	1.6 (1.0–2.6) ^c^
3Ab	–	80.9 (37.0–177.1)	11.4 (3.7–35.2)	12.2 (4.9–30.2)
2Ab	–	15.1 (10.0–22.9)	2.8 (1.2–6.8)	3.0 (1.7–5.3)
1Ab	–	2.1 (1.4–3.0)	0.9 (0.5–1.8) ^c^	–

^a^The serological criteria from the 2010 ACR/EULAR RA classification criteria, the concordance (Ccd.) of autoantibodies, and their combination without modification (2010 + Ccd.) and after deleting the 1Ab stratum (2010 + Ccd’)

^b^OR and their 95% confidence intervals

^c^This stratum did not contribute significantly to RA classification

**Fig. 1:**
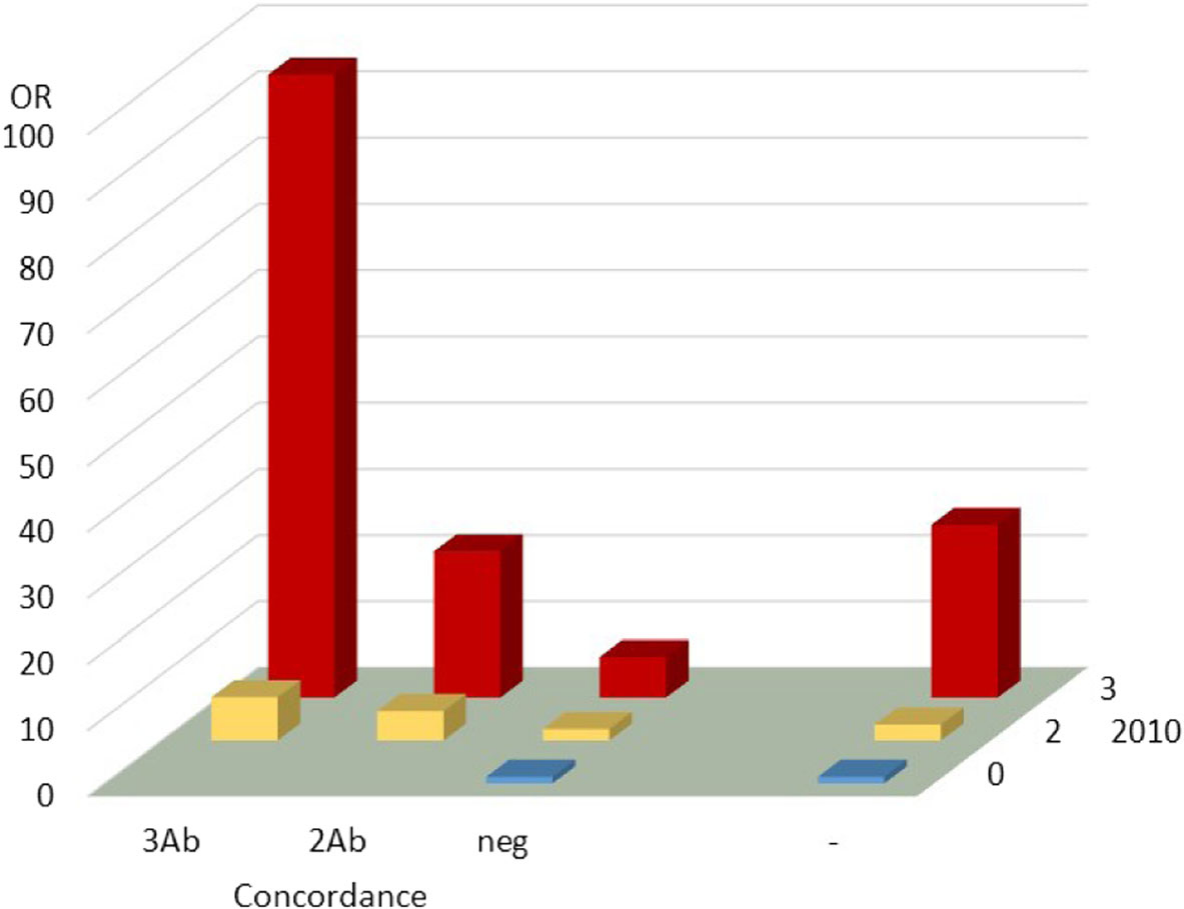
Odds ratio for RA classification corresponding to the EA patients stratified according to the combined 2010 ACR/EULAR and the concordance serological criteria

Once the contribution of the two types of criteria was demonstrated, the OR corresponding to the patients stratified simultaneously with the combined criteria was determined. The results were compared with the OR corresponding to the 2010 ACR/EULAR serological criteria (Fig. 1). The maximum OR (OR = 94.0, 95% CI = 40.7–217.2) was obtained with the patients that were simultaneously positive for the 3 antibodies and showed 3-points in the 2010 score. The patients with 3-points and 2 concordant antibodies followed (OR = 22.1, 95% CI = 13.5–36.0). This latter OR was slightly smaller than the corresponding to the 3-points score of the 2010 ACR/EULAR criteria. Therefore, only the group of patients combining the 3-points score and the concordance for the 3 antibodies required a higher weight than in the 2010 ACR/EULAR criteria.

It was also relevant to assess the overall performance of the different serological criteria. This evaluation was done determining the R^2^ and AIC of each model. The R^2^ estimates the fraction of the variance that is accounted by the criteria, whereas the AIC is proportional to the information loss taking into consideration the complexity of the model. The two measures were concordant in all the comparisons (Table 4). They showed that the performances of the 2010 ACR/EULAR serological criteria and the concordance criteria were almost identical (Table 4). In contrast, the combination of the two types of serological criteria explained a higher fraction of the variance and showed a lower AIC than the separate criteria. The difference was highly favorable to the combined models relative to the separate criteria, as the change in AIC was > 30 and a difference of 10 is already considered very convincing [14]. However, there were no differences between the combined criteria including or excluding the patients that were only positive for 1 antibody.

**Table 4:** Overall fit of the models with different serological criteria for RA classification

Model fit	2010 ACR/EULAR ^a^	Concordance	2010 + Ccdc.	2010 + Ccdc.’
*R*^2^	0.452	0.454	0.483	0.483
AIC	1033.3	1033.8	1001.8	999.9

^a^The serological criteria from the 2010 ACR/EULAR RA classification criteria, or based on the concordance of autoantibodies, and their combination without modification (2010 + Ccd.) and after deleting the 1Ab stratum (2010 + Ccd’)

As a final test, we also compared the classification performance of criteria that included the non-serological component together with each of the serological criteria. This analysis was only possible with a fraction of the patients (537 patients), but the results followed the above described: modest improvements with the alternative models (Table 5). The concordance criteria showed the same sensitivity than the 2010 ACR/EULAR criteria with a 2.7% increase in specificity, and the combined criteria brought a further increase of 1.1% in specificity together with a 1.8% improvement in sensitivity. These performance changes meant that 15 less patients were wrongly classified with the combined criteria than with the 2010 ACR/EULAR criteria of a total of 180 misclassified patients with the latter. As these results depend on the scores, we attributed to each level of autoantibodies, we also performed a comparison of the criteria without scores, directly from the logistic regression (Table 5). This analysis was adjusted to obtain 80% sensitivity with all the criteria. The results were similar, showing the same rank of specificities and accuracies. The improvement in specificity with the combined criteria over the 2010 ACR/EULAR criteria was 3.1% in this comparison.

**Table 5:** Performance of the classification criteria for RA with different serological components

Serological component	Scores ^a^			Logistic regression ^b^	
	Specificity	Sensitivity	Accuracy	Specificity	Accuracy
2010 ACR/EULAR	72.6	78.8	75.8	73.7	77.1
Concordance	75.3	78.8	77.1	74.9	77.8
Ccdc. + 2010	76.4	80.6	78.8	76.8	78.6

^a^Scores were as in the 2010 ACR/EULAR criteria; or depending on the number of concordant antibodies as 5 for 3, 3 for 2, and 1 for 1 antibodies; or combining the two criteria as 5 for 3 or 2 antibodies with high titres, 3 for 3 or 2 antibodies with low titres, and 1 for 1 antibody irrespective of the titres

^b^Classification was done with logistic regression including the non-serological and the serological criteria and adjusted to obtain 80% sensitivity

## Discussion

Our results have suggested a way to improve the RA classification of EA patients by incorporating the concordant presence of 3 RA autoantibodies. This approach is supported by various analyses. First, the higher PPV of the concordance for 3 or 2 antibodies than of the current serological component of the 2010 ACR/EULAR criteria. Second, the independent contributions of the concordance of antibodies and of the 2010 serological criteria to prediction models that combined them. Third, the increase in fit to the data of the combined prediction models relative to the current serological criteria. Fourth, the exploratory analysis showing improved performance of the classification criteria that included a combined serological component. These analyses also lead to the realization that a sizeable set of EA patients, in whom the three antibodies are present, deserve direct classification as RA.

These improvements affect precision and discrimination of the classification among EA patients. They are aspects of the classification outside the main focus of recent research, which has been placed in reducing “the seronegative gap” [17]. That is, in identifying autoantibodies that could serve as biomarkers in RA patients lacking RF and anti-CCP. However, the new RA autoantibodies that are best-established cover only a fraction of the seronegative gap [5, 13]. The gain in sensitivity afforded has not proved sufficient to compensate for the loss of specificity associated with increasing the number of alternative antibodies in EA patients [5–7]. Exactly this type of results led us and others to conclude that ACarPA did not contribute significantly to RA classification [6, 7]. Here, a change in perspective has shown the possibility of turning the concordance between RA antibodies into a source of useful information.

The insight leading us to consider antibody concordance as a potential classifier came from the demonstration of the high specificity of this phenotype [8]. In effect, the concordant presence of RF, anti-CCP, and ACarPA showed specificities of 98–100% in a set of 12 case-control studies comparing: RA patients before disease development to healthy controls, and RA patients after clinical onset to healthy controls, or healthy first-degree relatives, or diseased controls [8]. This high specificity, which was also observed in our EA patients (98.7%), led the authors to propose that triple antibody positivity could be used to identify individuals at risk of developing RA.

The most evident improvement that concordance of antibodies could provide is the direct classification of EA patients with 3 antibodies as RA. The PPV (96.1%) and OR (80.9) we have observed are sufficiently high as to propose this idea. The combination with the 2010 serological criteria was not necessary for this improvement, as it did not significantly modify classification at the top level.

In addition, the combined serological criteria permitted a more precise stratification of the RA prediction. For example, the high titres of RF or anti-CCP receive 3-points in the 2010 ACR/EULAR criteria, whereas with the combined criteria they were divided into three groups: those with 3 antibodies would be classified directly as RA (in the context of EA), the patients with 2 antibodies would remain in the 3-point level and the patients without concordant antibodies would receive a lower score. This detailed prediction led to the improvements reflected in the measures of model fit. Translating the increase in precision into practical benefit would require integration of the serological scoring with other clinical variables. According to the data obtained here, the serological scores in the future classification criteria will expand a larger range than currently. Predictably, they will include a top-level equivalent to RA classification provided that other criteria for EA are fulfilled and three or four lower scores. As an initial exploration, we set a four-level score for the serological component that increased its weight. It resulted in better performance of the classification than the obtained with the 2010 ACR/EULAR criteria. The same happened when the relative weights of each level were obtained from the logistic regression. A more definitive scoring system will require adjusting together the scores of the serological and non-serological components for optimal performance. Additional steps could be an assessment of the criteria by experts, as done with the 2010 ACR/EULAR criteria, and validation in independent EA patients including patients with any joint swelling, in place of the two swollen joints required in our EA cohorts.

We restricted this study to explore the potential benefit of concordance between RF, ACPA, and ACarPA because of its novelty and the need to decide if ACarPA could have any role in the classification of RA patients. However, there is strong evidence indicating that ACPA has more predictive value than RF (a feature also observed in our EA patients) and it could be possible to improve classification by differentiating between them. Also, the concordant presence of RF and anti-CCP could be used to improve classification without the need for new antibody determinations to the clinic. The PPV of the two-antibodies concordance in our patients was 93.1% and the OR = 43.9, which are higher values than those obtained with the 2010 serological criteria. These approaches based on the concordance of RF and anti-CCP or in differentiated scores for RF and anti-CCP will improve classification to a lesser degree, but do not require new antibody determinations beyond the widely available in the clinic. Finally, it is possible that other autoantibodies, different from the three considered here, could produce improvements in RA classification [5, 17].

## Conclusions

Our results have shown the possibility of improving the discrimination and precision of the serological component in the RA classification of EA patients. Provided that our results are replicated, and extended to patients with one swollen joint, the top-level corresponding to the presence of the 3 antibodies will be sufficient for RA classification in the EA context. This step by itself could increase the number of patients receiving appropriate classification with a low fraction of false positives. However, the full advantage of the predictive power of antibody concordance will require modification of the weights given to each stratum of RA patients in the whole classification criteria.

## Data Availability

The datasets used and/or analyzed during the current study are available from the corresponding author on reasonable request.

## Abbreviations

1Ab: One antibody
2Ab: Two antibodies
3Ab: Three antibodies
95% CI: 95% confidence interval
ACarPA: Anti-Carbamylated Protein Antibodies
ACR: American College of Rheumatology
AIC: Akaike’s Information Criterion
Anti-CCP: Anti-Citrullinated Protein Antibodies
Ccd: Concordance
DMARD: Disease-Modifying Anti-Rheumatic Drugs
EA: Early arthritis
ELISA: Enzyme-linked immunosorbent assay
EULAR: European League Against Rheumatism
IdiPAZ: Instituto de Investigacion Hospital Universitario La Paz
OR: Odds ratio
PEARL: Princesa Early Arthritis Register Longitudinal
PPV: Positive predictive value
RA: Rheumatoid arthritis
RF: Rheumatoid factor
ULN: Upper limit of normal

## References

[1] Smolen JS, Landewe R, Bijlsma J, Burmester G, Chatzidionysiou K, Dougados M, Nam J, Ramiro S, Voshaar M, van Vollenhoven R (2017). EULAR recommendations for the management of rheumatoid arthritis with synthetic and biological disease-modifying antirheumatic drugs: 2016 update. Ann Rheum Dis.

[2] Aletaha D, Neogi T, Silman AJ, Funovits J, Felson DT, Bingham CO, Birnbaum NS, Burmester GR, Bykerk VP, Cohen MD (2010). 2010 Rheumatoid arthritis classification criteria: an American College of Rheumatology/European league against rheumatism collaborative initiative. Arthritis Rheum.

[3] Radner H, Neogi T, Smolen JS, Aletaha D (2014). Performance of the 2010 ACR/EULAR classification criteria for rheumatoid arthritis: a systematic literature review. Ann Rheum Dis.

[4] Hua C, Daien CI, Combe B, Landewe R (2017). Diagnosis, prognosis and classification of early arthritis: results of a systematic review informing the 2016 update of the EULAR recommendations for the management of early arthritis. RMD open.

[5] Juarez M, Bang H, Hammar F, Reimer U, Dyke B, Sahbudin I, Buckley CD, Fisher B, Filer A, Raza K (2016). Identification of novel antiacetylated vimentin antibodies in patients with early inflammatory arthritis. Ann Rheum Dis.

[6] Regueiro C, Nuno L, Ortiz AM, Peiteado D, Villalba A, Pascual-Salcedo D, Martinez-Feito A, Gonzalez-Alvaro I, Balsa A, Gonzalez A (2017). Value of measuring anti-Carbamylated protein antibodies for classification on early arthritis patients. Sci Rep.

[7] Boeters DM, Trouw LA, van der Helm-van Mil AHM, van Steenbergen HW (2018). Does information on novel identified autoantibodies contribute to predicting the progression from undifferentiated arthritis to rheumatoid arthritis: a study on anti-CarP antibodies as an example. Arthritis Res Ther.

[8] Verheul MK, Bohringer S, van Delft MAM, Jones JD, Rigby WFC, Gan RW, Holers VM, Edison JD, Deane KD, Janssen KMJ (2018). Triple positivity for anti-Citrullinated protein autoantibodies, rheumatoid factor, and anti-Carbamylated protein antibodies conferring high specificity for rheumatoid arthritis: implications for very early identification of at-risk individuals. Arthritis Rheumatol.

[9] Gonzalez-Alvaro I, Ortiz AM, Alvaro-Gracia JM, Castaneda S, Diaz-Sanchez B, Carvajal I, Garcia-Vadillo JA, Humbria A, Lopez-Bote JP, Patino E (2011). Interleukin 15 levels in serum may predict a severe disease course in patients with early arthritis. PLoS One.

[10] Orozco G, Pascual-Salcedo D, Lopez-Nevot MA, Cobo T, Cabezon A, Martin-Mola E, Balsa A, Martin J (2008). Auto-antibodies, HLA and PTPN22: susceptibility markers for rheumatoid arthritis. Rheumatology.

[11] Arnett FC, Edworthy SM, Bloch DA, McShane DJ, Fries JF, Cooper NS, Healey LA, Kaplan SR, Liang MH, Luthra HS (1988). The American rheumatism association 1987 revised criteria for the classification of rheumatoid arthritis. Arthritis Rheum.

[12] Montes A, Regueiro C, Perez-Pampin E, Boveda MD, Gomez-Reino JJ, Gonzalez A (2016). Anti-Carbamylated protein antibodies as a reproducible independent type of rheumatoid arthritis autoantibodies. PLoS One.

[13] Shi J, Knevel R, Suwannalai P, van der Linden MP, Janssen GM, van Veelen PA, Levarht NE, van der Helm-van Mil AH, Cerami A, Huizinga TW (2011). Autoantibodies recognizing carbamylated proteins are present in sera of patients with rheumatoid arthritis and predict joint damage. Proc Natl Acad Sci U S A.

[14] Anderson DR, Burnham K (2004). Model selection and multi-model inference: a practical information-theoretic approach.

[15] R: A Language and Environment for Statistical Computing. http://www.R-project.org. Accessed 15 Apr 2019.

[16] The jamovi project: *jamovi*. In*.*, Version 0.9 edn; 2019: Computer software.

[17] Trouw LA, Mahler M (2012). Closing the serological gap: promising novel biomarkers for the early diagnosis of rheumatoid arthritis. Autoimmun Rev.

